# An automated surgical decision-making framework for partial or radical nephrectomy based on 3D-CT multi-level anatomical features in renal cell carcinoma

**DOI:** 10.1007/s00330-023-09812-9

**Published:** 2023-06-08

**Authors:** Huancheng Yang, Kai Wu, Hanlin Liu, Peng Wu, Yangguang Yuan, Lei Wang, Yaru Liu, Haoyang Zeng, Junkai Li, Weihao Liu, Song Wu

**Affiliations:** 1 Teaching Center of Shenzhen Luohu Hospital, Shantou University Medical College, Shantou, 515000 China; 2 Department of Urology, The Third Affiliated Hospital of Shenzhen University (Luohu Hospital Group), Shenzhen, 518000 China; 3 Shantou University Medical College, Shantou University, Shantou, 515000 China; 4 Shenzhen Following Precision Medical Research Institute, Luohu Hospital Group, Shenzhen, 51800 China; 5 Department of Radiology, The Third Affiliated Hospital of Shenzhen University (Luohu Hospital Group), Shenzhen, 518000 China; 6 Department of Urology, South China Hospital, Health Science Center, Shenzhen University, Shenzhen, 518116 China

**Keywords:** Carcinoma, renal cell, Machine learning, Deep learning, Decision-making, Nephrectomy

## Abstract

**Objectives:**

To determine whether 3D-CT multi-level anatomical features can provide a more accurate prediction of surgical decision-making for partial or radical nephrectomy in renal cell carcinoma.

**Methods:**

This is a retrospective study based on multi-center cohorts. A total of 473 participants with pathologically proved renal cell carcinoma were split into the internal training and the external testing set. The training set contains 412 cases from five open-source cohorts and two local hospitals. The external testing set includes 61 participants from another local hospital. The proposed automatic analytic framework contains the following modules: a 3D kidney and tumor segmentation model constructed by 3D-UNet, a multi-level feature extractor based on the region of interest, and a partial or radical nephrectomy prediction classifier by XGBoost. The fivefold cross-validation strategy was used to get a robust model. A quantitative model interpretation method called the Shapley Additive Explanations was conducted to explore the contribution of each feature.

**Results:**

In the prediction of partial versus radical nephrectomy, the combination of multi-level features achieved better performance than any single-level feature. For the internal validation, the AUROC was 0.93 ± 0.1, 0.94 ± 0.1, 0.93 ± 0.1, 0.93 ± 0.1, and 0.93 ± 0.1, respectively, as determined by the fivefold cross-validation. The AUROC from the optimal model was 0.82 ± 0.1 in the external testing set. The tumor shape Maximum 3D Diameter plays the most vital role in the model decision.

**Conclusions:**

The automated surgical decision framework for partial or radical nephrectomy based on 3D-CT multi-level anatomical features exhibits robust performance in renal cell carcinoma. The framework points the way towards guiding surgery through medical images and machine learning.

**Clinical relevance statement:**

We proposed an automated analytic framework that can assist surgeons in partial or radical nephrectomy decision-making. The framework points the way towards guiding surgery through medical images and machine learning.

**Key Points:**

*• The 3D-CT multi-level anatomical features provide a more accurate prediction of surgical decision-making for partial or radical nephrectomy in renal cell carcinoma.*

*• The data from multicenter study and a strict fivefold cross-validation strategy, both internal validation set and external testing set, can be easily transferred to different tasks of new datasets.*

*• The quantitative decomposition of the prediction model was conducted to explore the contribution of each extracted feature.*

**Supplementary Information:**

The online version contains supplementary material available at 10.1007/s00330-023-09812-9.

## Introduction

Renal cell carcinoma (RCC), which is the most common type and represents 90% of all kidney cancer, accounts for 3% of all cancers and is by far the highest incidence occurring in Western countries [1, 2]. Typically, radical nephrectomy (RN) and partial nephrectomy (PN) are the two main surgical treatment options for RCC. In the past four decades, RN was the standard treatment for RCC. With the improvement of modern surgical techniques, PN is considered the most appropriate surgical treatment for localized RCC [3–5]. PN offers a faster recovery and protection of renal function compared to RN, thereby reducing the risk of cardiovascular or metabolic disease after surgery [6–8].

Computed tomography (CT) plays an important role throughout the RCC patient pathway, from screening, diagnosis, and staging to treatment and assessment [9]. CT/CTA is the current indispensable standard in the evaluation of surgical approaches for kidney cancer, which can evaluate the vascular, renal, and tumor anatomy and provide a basis for preoperative planning. According to the National Comprehensive Cancer Network Guidelines published in 2021, patients with high p–T and high p-G often undergo RN [10]. For the optimal outcomes of RCC surgery, three variables (margin-ischemia-complications (MIC)) are taken into account: (1) surgical margins are negative, (2) warm ischemia time is < 20 min, and (3) there are no major complications [11, 12]. The higher the pathological T-stage (p–T), the more difficult the surgery will be and more difficult it is to meet MIC criteria [13]. When the pathological grade (p-G) is high, the boundary between the tumor and the kidney is unobvious, and the tumor is more difficult to cut cleanly [14]. Conventional imaging can only predict clinical T-stage (c-T), which is related to physician experience and cannot accurately diagnose p–T and p-G.

Recently, machine learning algorithm has confirmed their ability to predict p–T and p-G by CT features for RCC [15–17]. Although these studies present outstanding performance in methodological metrics, there are still two restrictions. For one thing, the model relies on manually annotated regions of interest (ROIs) by specialists, which is unusable under most circumstances [18, 19]. For another, subject to the “black box” traits which lacks explanatory research and acts as “black box” of deep learning algorithm [20, 21], it is difficult to correctly interpret the decision-making process within the model, so the clinicians are afraid to use it.

To the best of our knowledge, there was no literature reporting the value of machine learning–based CT features for RCC surgical approach. Considering that surgical decisions for RCC are complex, we need a more comprehensive collection of anatomical features for surgical approach prediction. In this study, we integrate the traditional radiomic features, p–T and p-G staging features, and the whole ROI anatomical features to construct an automated surgical decision-making framework for partial or radical nephrectomy in RCC. Besides, we also quantitatively analyzed the impact of extracted features on model decisions through the SHapley Additive exPlanations (SHAP) value to elucidate how the decisions are made.

## Materials and methods

### Participant cohorts

This is a retrospective study based on multi-center cohorts with kidney cancer. The internal data set contains the participants who underwent nephrectomy from five open-source data sets (CPTCA-CCRCC, TCGA-KIRC, TCGA-KIRP, TCGA-KICH, and C4KC-KiT from The Cancer Imaging Archive) and two local hospitals from 2020 to 2022 (The Eighth Affiliated Hospital of Sun Yat-sen University and Sun Yat-sen University Cancer Center). The external testing set contains the participants who underwent nephrectomy in another local hospital (The Third Affiliated Hospital of Shenzhen University) between 2020 and 2022. This study was approved by the local institutional review board (KY2022-036–01). Informed consent documents are waived for this retrospective analysis used anonymous clinical data and images.

### Data preparation and image segmentation

**Fig. 1:**
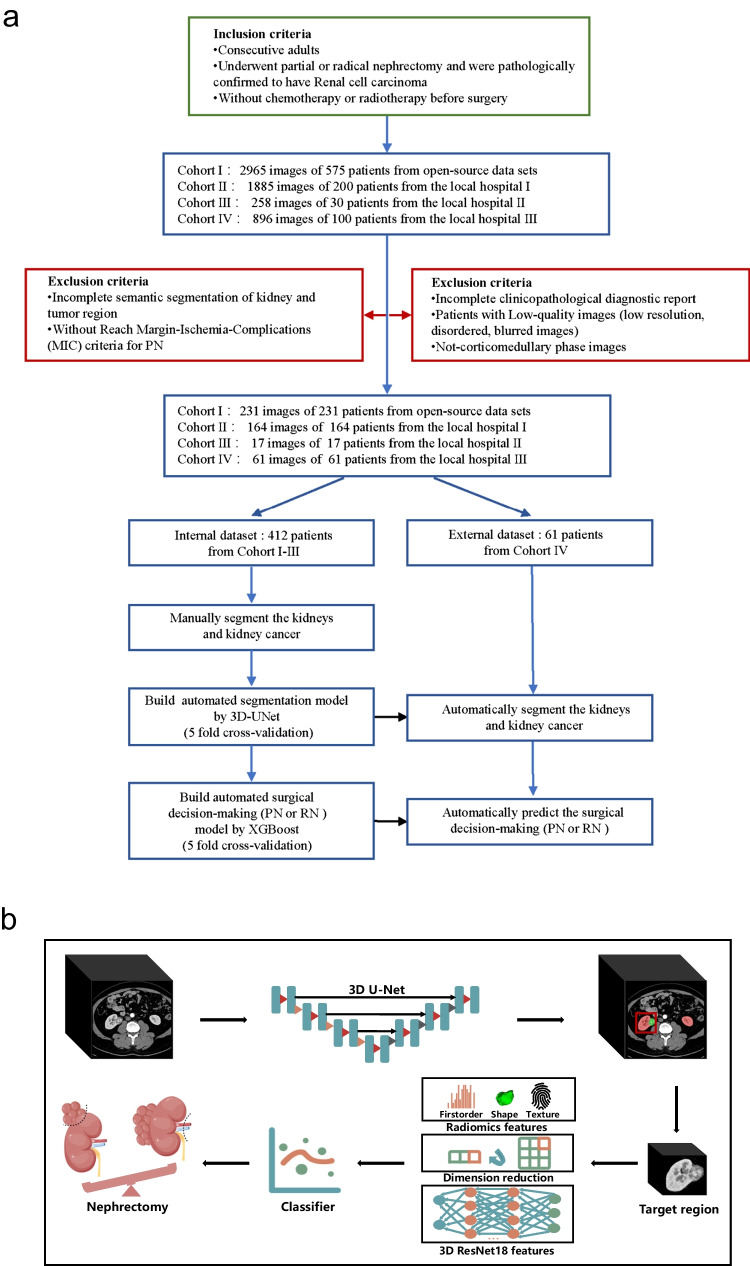
Analytical framework. **a** Flow diagram. Showing initial numbers of participants in open-source data sets (CPTCA-CCRCC, TCGA-KIRC, TCGA-KIRP, TCGA-KICH, and C4KC-KiT from The Cancer Imaging Archive) and there local hospitals’ data and the reasons for patient exclusion. **b** The surgical decision-making framework for partial or radical nephrectomy based on 3D-CT multi-level anatomical features in renal cell carcinoma (some material from *Figdraw*). A 3D-UNet model (top) is used to identify and segment lesions and extracted features from multi-level are utilized for prediction and classification (bottom)

Additional quality selection was conducted to exclude cases with low resolution or incomplete clinical information, and only keep the corticomedullary phase images. In this study, we strictly abide by the following inclusion and exclusion criteria (see the supplementary file for details and Fig. 1a). *Inclusion criteria*: (1) consecutive adults; (2) underwent partial or radical nephrectomy and were pathologically confirmed to have renal cell carcinoma; (3) without chemotherapy or radiotherapy before surgery. *Exclusion criteria*: (1) incomplete semantic segmentation of kidney and tumor region; (2) without reach MIC criteria for PN [11, 12]; (3) incomplete clinicopathological diagnostic report; (4) patients with low-quality images (low resolution, disordered, blurred images); (5) not-corticomedullary phase images. After data preparation, the corticomedullary phase images were manually annotated and segmented with kidneys and kidney cancer by two radiologists (with more than 10 years of experience) and two well-trained medical students. They were blinded to the pathological and surgical data. Using 3D images and segmentation results, an automatically kidneys and kidney cancer segmentation model was constructed by a 3D-UNet based network [22]. Hyperparameters, such as some pooling operations, batch size, and patch size, were selected based on the properties of the dataset. And then, the prediction results of segmentation model were checked and amended by a specialist (with more than 20 years of experience) to ensure the accuracy of kidney and cancer boundaries.

### Multi-level feature extraction

**Fig. 2:**
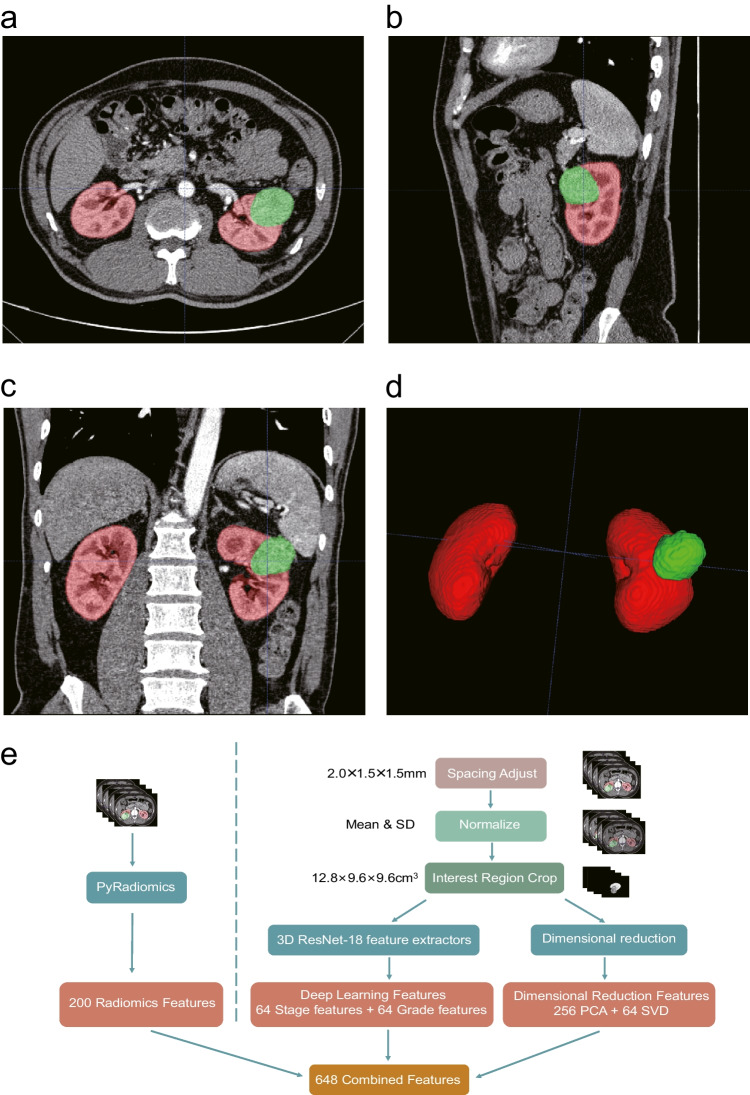
CT images after automatic segmentation by 3D-UNET and the multi-level features extraction solution. **a**–**d** A case in external dataset: male, 58, T1bN0M0, grade = 2, ground truth surgery underwent partial nephrectomy, the surgical decision-making framework also recommend partial nephrectomy (**a** axial image; **b** sagittal image; **c** coronal image; **d** 3D image). **e** 200 Radiomics features were integrated with 128 deep learning features and 320 dimension reduction features as the 648 combined features to the classifier

To fully extract the 3D anatomical features, a multi-level feature extraction solution was used in this study (Fig. 2c). Our multi-level features include (1) 200 radiomics features, which contains the texture, morphological, and statistical features about kidneys and kidney cancer, were calculated by PyRadiomics (v3.0.1); (2) 128 features, which are the task-oriented and reflects the high-tumor and low-tumor stage and grade characteristic in the pathology, were extracted by a trained 3D ResNet-18 neural network; (3) 320 features by dimensionality reduction of ROI, which represents the original voxel information, were performed by principal component analysis (PCA) and singular value decomposition (SVD). Moreover, we were able to predict clinical stage and pathological grade for two feature extractors. The area under the receiver operating characteristic curve (AUROC) was 0.74 ± 0.1 for T1/T2 vs. T3/T4 prediction (Fig. S2c) and 0.73 ± 0.1 for G1/G2 vs. G3/G4 prediction (Fig. S2d) on the testing set. After experiments, the multi-level features enable the classifier to achieve the optimum performance. The technical details for multi-level feature extraction can be found in the supplementary materials.

### Partial versus radical nephrectomy prediction

Gradient boosting decision tree (XGBoost, v1.3.3) was used to predict the surgical decision-making (partial versus radical nephrectomy) [23]. The fused multi-level features are used as the input for the model. For effectiveness testing and model selection, fivefold cross-validation was applied in the internal training set. And the above five models were further tested on the external data set. To explore the process of model decision-making, SHAP values were used to decompose the model decision into individual feature influences [24]. A high SHAP value shows that the feature affects the essence of the model decision. All these statistical analyses and experiments were conducted in python (v3.8) and R (v3.6.3). The statistical analysis is of significance when the *p* value was < 0.05. The uncertainty of the estimate such as accuracy, AUROC was quantified at a 95% confidence interval.

## Results

### Participant characteristics

CT images with clinical, pathological, and surgical information were collected and selected from open-source cohorts and local hospitals’ cohorts. In the initial stage, a total of 875 cases with 5510 CT scan images were included (see the supplementary file for details). After image screening (Fig. S1), 473 corticomedullary phase images from 473 participants (190 females and 283 males) were involved in the following analysis. The basic and clinical information of these participants are shown in Table 1. The mean age (standard deviation) is 56.3 (13.3) years. For efficient model evaluation and selection, we adopt fivefold cross-validation method on the internal data set (412 cases). In each fold, there are 335 cases (80%) in the training set and 77 cases (20%) in the validation set. The external testing set with 61 cases was used for the final testing of our proposed model. The diagram of the automated surgical decision-making framework is shown in Fig. 1.

**Table 1:** Basic, clinical, and pathologic characteristics of patients

Characteristic	Value
Participants	473
Age(year)	56.3 ± 13.3
Female	190
Male	283
Histologic subtype	
Clear cell renal cell carcinoma	356
Chromophobe renal cell carcinoma	39
Papillary renal cell carcinoma	38
Oncocytoma	17
Collecting duct carcinoma	9
Renal medullary carcinoma	7
Renal carcinoma associated with Xp11.2 translocation	7
Pathologic tumor stage	
T1	201
T2	146
T3	82
T4	32
Other	12
Pathologic tumor grace	
G1	61
G2	187
G3	120
G4	26
N/A	79
Nephrectomy	
Partial nephrectomy	240
Radical nephrectomy	202
N/A	31

### Multi-level features provide the best performance

**Fig. 3:**
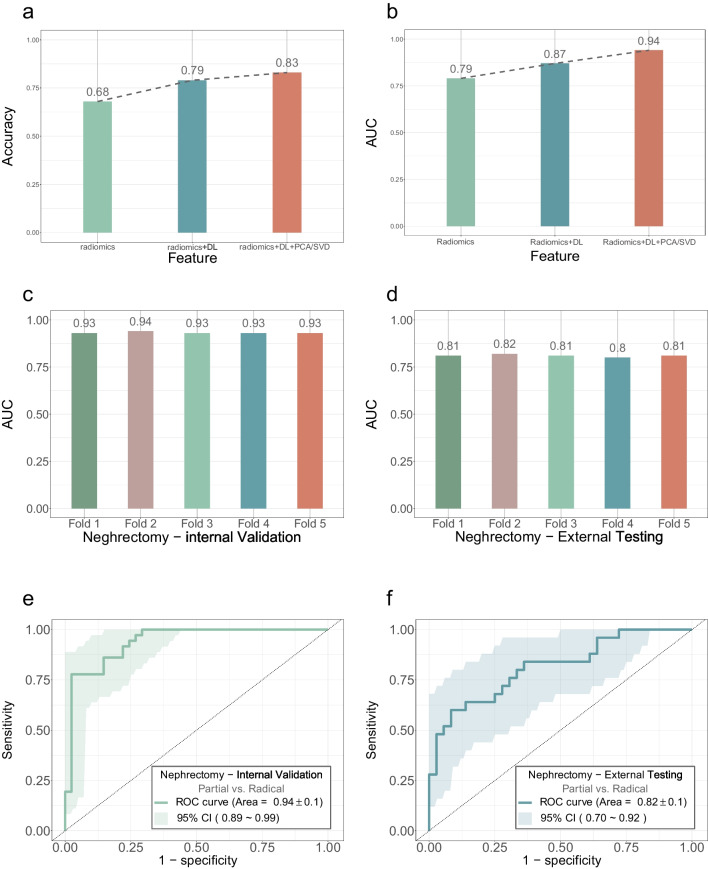
The analysis and results of multi-angle feature extraction and performance of proposed feature prediction model. **a**, **b** The accuracy and AUROC of different feature extraction solution. **c** The AUROC of surgical approach prediction model in internal validation set by the fivefold cross-validation. **d** The AUROC of surgical approach prediction model in external testing set by the fivefold cross-validation. **e**The AUROC of surgical approach prediction model in internal validation set. **f** The AUROC of the surgical approach prediction model in external testing set

The ROI for outlining the entire 3D kidney and tumor were automatically segmented by a 3D-UNet-based convolutional neural network (Fig. 2a, b). The multi-level features were extracted by using the ROI and original images. A pre-experiment was conducted to select the best combinations of multi-level features. In the task of partial vs. radical nephrectomy prediction, the AUROC was 0.79 ± 0.1, 0.87 ± 0.1, and 0.94 ± 0.1 when using radiomics features only, the two-level merged features, and the multilevel features as feature inputs, while the accuracy was 52 ± 9.8%,74 ± 8.6%, and 88 ± 6.4% at 95% confidence level, respectively (Fig. 3a, b). The combination of multi-level features achieved the best performance in the classification task. The multi-level features extraction solution will provide the more comprehensive knowledge.

### The automatic framework presents robust analytical capability

For the internal validation, the AUROC of the partial vs. radical nephrectomy prediction was 0.93 ± 0.1, 0.94 ± 0.1, 0.93 ± 0.1, 0.93 ± 0.1, and 0.93 ± 0.1 by the fivefold cross-validation, respectively (Fig. 3c). For the external testing, the AUROC was 0.81 ± 0.1, 0.82 ± 0.1, 0.81 ± 0.1, 0.8 ± 0.1, and 0.81 ± 0.1 by the fivefold cross-validation, respectively (Fig. 3d). The AUROC from the optimal model was 0.94 ± 0.1 and 0.82 ± 0.1 for partial vs radical nephrectomy prediction at the internal validation set (Fig. 3e) and external testing set (Fig. 3f), respectively.

### Feature contribution evaluation by SHAP values

**Fig. 4:**
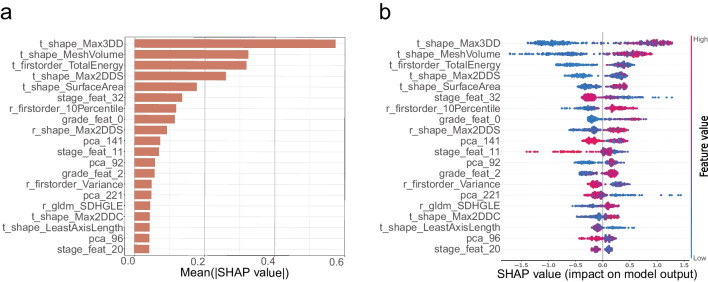
Ranking of SHAP values for the explanation of machine learning classification. **a**, **b** Barplot and beeswarm plot display the SHapley Additive exPlanations (SHAP) values for the training set of surgical approach prediction model

To explore the contribution of features to model prediction, the SHAP values of each feature for each sample was calculated. Based on the SHAP values, the top 20 contributors for partial vs radical nephrectomy were shown on the on beeswarm plots (Fig. 4a) and bar plots (Fig. 4b). The positive SHAP values indicate a higher likelihood for the corresponding prediction. For the bar plots, the shape-related features such as t_shape_Max3DD (tumor shape Maximum 3D Diameter) played the most vital role in the model decision, which conforms to the criteria of tumor size in clinical practice guidelines [25, 26] of kidney cancer referring to predict partial vs radical nephrectomy (see the supplementary file for details). Moreover, the extracted features by deep learning (such as stage_feat_32 and grade_feat_0) and dimensionality reduction (such as pca_141) also participate in the model prediction.

## Discussion

More than 10 years ago, PN surgical scoring system such as RENAL [25] and PADUA [26] has been widely used in clinical practice; there are certain limitations. More than 20 different scoring systems, mostly based on 2D CT or MRI, have been applied to open and laparoscopic surgery [27]. There is a contradiction between complexity and performance in the current scoring systems. Multi-parameter assessment system works well but complex and cumbersome to operate, while few-parameter assessment system is convenient to use but with poor stability [28]. Therefore, there is an urgent need for a practical, automated preoperative assessment system to enable more accurate, efficient, and reproducible assessments.

To move beyond this limitation, we constructed an automated analytical system for both image preprocessing, RCC localization, multi-level feature extraction, and partial vs. radical nephrectomy prediction. Although many studies attempted to predict clinical properties by radiomics, they required too much manpower for ROI segmentation and were not comparable to our work [16]. Unlike other studies that extracted only 2D features at the tumor center [18, 19], this work performed more comprehensive multi-level feature extraction based on 3D ROI. Radiomics provides an extremely efficient tool for quantitative feature extraction that converts medical images into shape base and morphological and statistical data about ROI. By the powerful ability, radiomics features were used to develop prognostic and diagnostic models and promotes the exponential growth of medical image analysis [29–33]. But the degree of automation and standardization of radiomics is low, and there is still much room for improvement in the accuracy and robustness of prediction results [34]. All steps of deep learning (segmentation, feature extraction, modeling) performed separately and sequentially are performed by neural network [35, 36]. These methods are data-hungry and therefore datasets much larger than those usually available in radiomics studies are needed for efficient training [35, 36]. Therefore, we combined two techniques making use of their complementary value in order to build more efficient and automated predictive models.

As we know, feature selection plays a vital role in model prediction. For surgical options in RCC, patients with high p–T and high p-G often have difficulty meeting MIC criteria, so RN is often selected [13]. Therefore, to incorporate tumor infiltration and malignancy into predicting the surgical approach, two feature extractors based on 3D ResNet-18 were established with stage and grade as labels respectively. At the same time, to dig the anatomical features, dimensionality reductions called PCA and SVD were used for the target region. We found that the multi-level features, processed by the above three methods, are more effective than the single one (Fig. 3a–b).

In most studies, the proposed machine learning models lacked explanatory research and act as “black box” [20, 21]. In application scenarios, a reliable model should not only adapt itself to any given dataset but also output an interpretable result. To this end, we quantitatively analyzed the relationship between the multi-level features and the model decision by SHAP values. Compared with other explanatory methods, such as class activation map [37], SHAP value can enumerate the influence of each feature for both individual case and the all dataset, which is more convincing for understanding model decision-making and even the causes for misclassifications. In Fig. 4, we can see that the radiomics features, such as tumor shape Maximum 3D Diameter contributed most to the partial vs. radical nephrectomy prediction, which is consistent with the knowledge that tumor size is the main factor in the most PN surgical scoring systems [27]. And the extracted features by deep learning (such as stage_feat_32 and grade_feat_0) and dimensionality reduction (such as pca_141) also participate in the model prediction. These results suggest that the multi-level features play a complementary role and improve model performance in partial vs. radical nephrectomy prediction (Fig. 3e–f).

The strategy of multi-level anatomical feature extraction can be easily transferred to different tasks of new datasets. Both the internal training and external testing set are heterogeneous which contain six open-source cohorts and three local  hospitals’ cohorts, respectively. Even if overfitting exists, the AUROC still reached 0.82 ± 0.1 for partial vs radical nephrectomy prediction at the external testing set (Fig. 3f). Besides, we performed a fivefold cross-validation to test the generalization ability and internal and external robustness of the model. Therefore, we consider the model overfitting to be acceptable and the model performance is outstanding and quite stable in such a heterogeneous dataset.

This work still has several limitations that require further improvement. Firstly, this was a retrospective and multicenter study, resulting in greater data heterogeneity, harder model training, and higher overfitting. Our framework will perform better in a larger and unified surgical standards data set. Secondly, the proposed model was limited to the analysis of renal cell carcinoma. The cases with benign renal lesions such as angiomyolipoma and renal adenoma were scarce in this study and the model predictions for benign disease were not tested. Thirdly, this work focused on partial vs. radical nephrectomy prediction and was based on the retrospective medical records. We do not know if it will improve patient outcomes. To achieve it, long-term follow-up results and further prospective studies are needed.

In conclusion, our study demonstrates the potential for partial vs. radical nephrectomy recommendation through CT images by machine learning. We proposed an automated analytic framework for accurate kidney cancer localization and multi-level anatomical feature extraction for 3D corticomedullary phase CT. In addition, we confirmed that the use of multi-level features can greatly improve model performance. We believe this research points the way towards guiding surgery through medical images and machine learning.

### Supplementary Information

Below is the link to the electronic supplementary material.

Supplementary file1 (PDF 706 KB)

## Data Availability

All of the code generated or used during the study are available in the GitHub repository (https://github.com/tiaAI/kidney_cancer_surgery_model_project-main.git). The original images and data used in this study are available from the corresponding author by request.

## Abbreviations

AUROC: Area under the receiver operating characteristic curve
PN: Partial nephrectomy
RCC: Renal cell carcinoma
RN: Radical nephrectomy
ROC: Receiver operating characteristic
ROI: Regions of interest
SHAP: The Shapley Additive Explanations
vs.: Versus

## Declarations

### Guarantor

The scientific guarantor of this publication is Prof. Song Wu.

### Statistics and biometry

Kai Wu majored in statistics and computer sciences, and has significant statistical expertise.

### Study subjects or cohorts overlap

All the cohorts have been previously reported in The Cancer Imaging Archive (TCIA), which is an open-source database and hosts a large archive of medical images accessible for public download. As far as we know, seldom researches integrated and analyzed all the kidney-cancer cohorts provided by TCIA. In this study, we proposed an analytical procedure by using these CT images, and firstly reported the automated surgical decision framework for partial or radical nephrectomy based on 3D CT multi-level anatomical features in renal cell carcinoma.

### Methodology

**• **retrospective

**• **surgical decision-making study/observational

**• **multicentre study
