# Supplementary material for: An automated surgical decision-making framework for partial or radical nephrectomy based on 3D-CT multi-level anatomical features in renal cell carcinoma
Source: Eur Radiol. 2023 Jun 8;33(11):7532–41. doi: 10.1007/s00330-023-09812-9 (PMC10598088; doi:10.1007/s00330-023-09812-9)
Supplement: Supplementary file 1 — Supplementary file1 (PDF 706 KB) [file 330_2023_9812_MOESM1_ESM.pdf]

An automated surgical decision-making framework for partial or radical nephrectomy based on 3D-CT multi-level anatomical features in renal cell carcinoma

ELECTRONIC SUPPLEMENTARY MATERIAL

Supplementary Figure

Center	Tube voltage (kV)	Tube current (mA)	Matrix	Slice thickness (mm)	Pitch	Image acquisition time (m)
Open-source data sets	NA	NA	512 × 512	5 or 10	NA	NA
Local hospital 1	120	200	512 × 512	1 ,2 or 5	0.87	30
Local hospital 2	120	360	512 × 512	1 ,2 or 5	0.98	35
Local hospital 3	120	280	512 × 512	1 ,2 or 5	0.89	32

Table.S1 CT sequences and acquisition for patients.

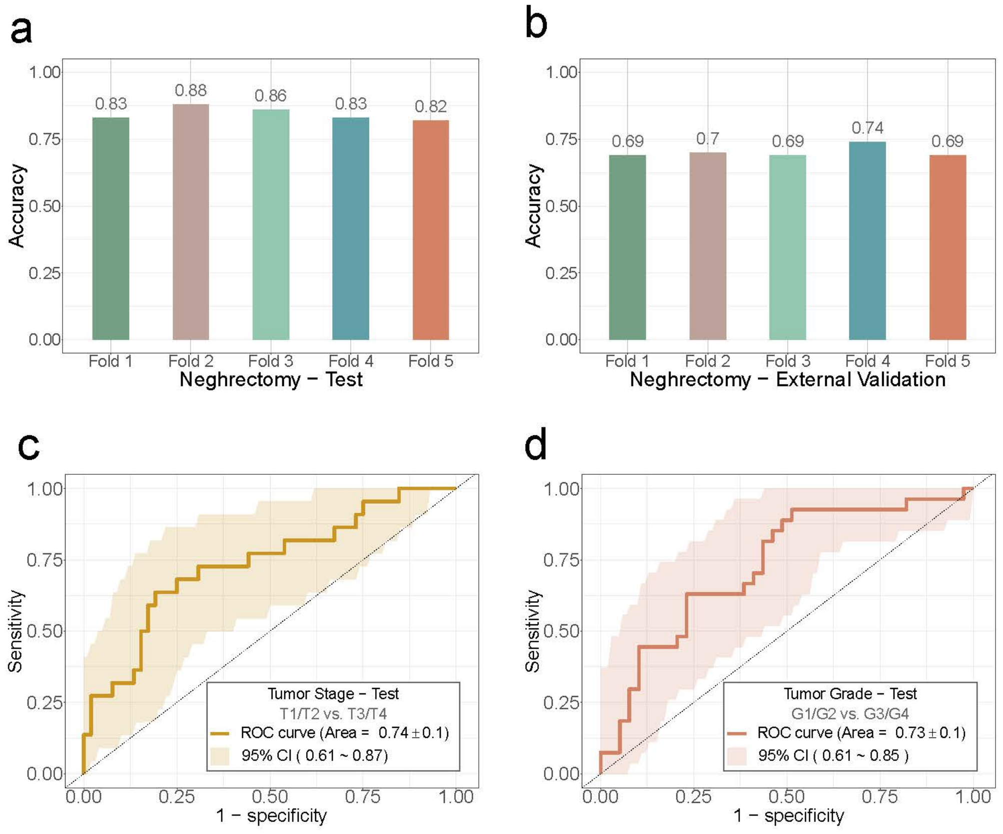

Fig.S1

(a) The Accuracy of surgical approach prediction model in internal validation set by the 5-Fold Cross-Validation..

(b) The Accuracy of surgical approach prediction model in external testing set by the 5-Fold Cross-Validation.

The AUROC of surgical approach prediction model in internal validation set by the 5-Fold Cross-Validation.

(c-d) Receiver operating characteristic (ROC) curve of the feature extractors in tumor stage T1&T2 vs. T3&T4 prediction (AUC = 0.74±0.1,0.61-0.87) and tumor grade G1&G2 vs. G3&G4 prediction (AUC = 0.73±0.1,0.61-0.85) at the 95%confidence level.

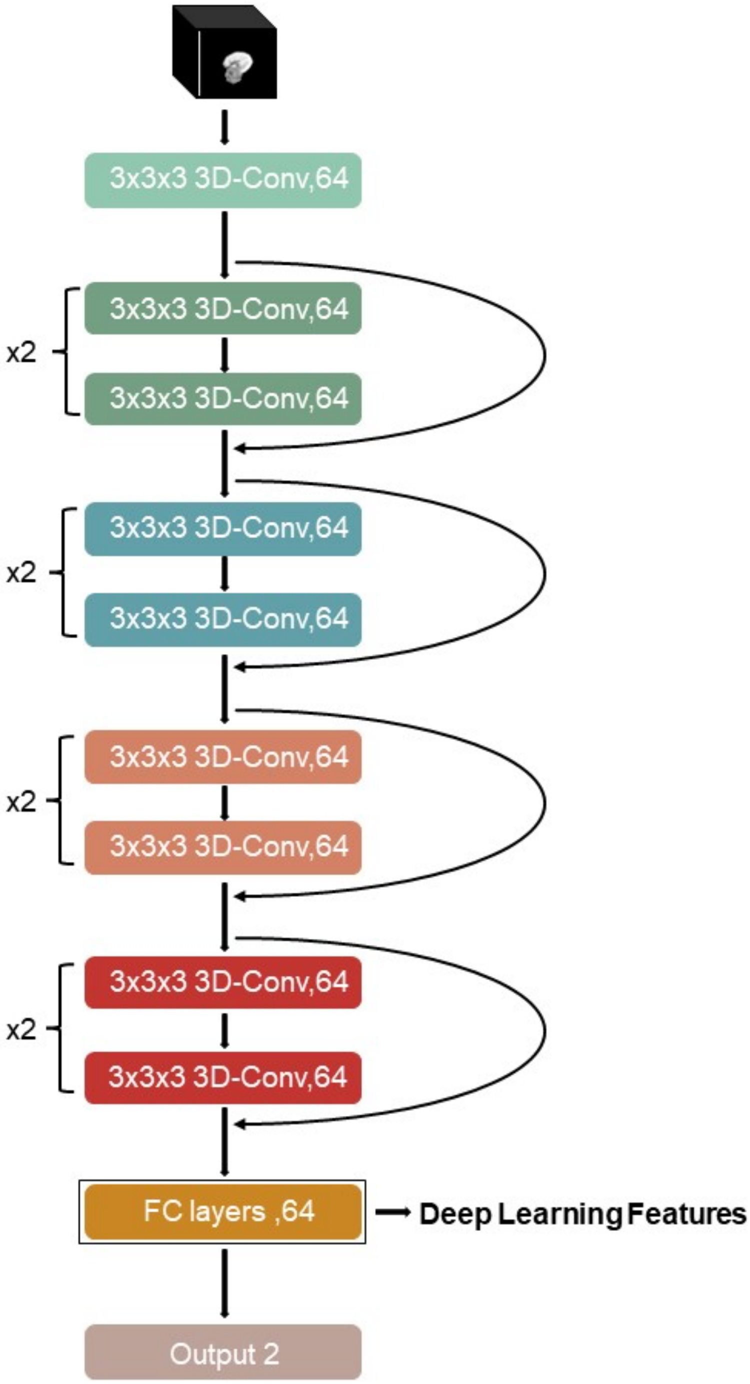

Fig.S2 Network structure of 3D ResNet-18

Eur Radiol (2023) Yang H, Wu K, Liu H et al.

Supplementary Method

CT scanning protocol

The CT image scanning was performed using a dual-source spiral CT system(SOMATOM Force, Siemens Medical System, Forchheim, Germany) in local hospital Ⅰ. The CT image scanning also was performed using a dual-source spiral CT system(SOMATOM Force, Siemens Medical System, Forchheim, Germany) in local hospital Ⅱ. The local hospital Ⅲ underwent CT scanning with a 128-slice spiral CT system (Brilliance iCT, Philips System). The CT protocol data of five open-source data sets was missing. The CT scanning parameters were showed in Table S1.

Participant data

Five cohorts including C4KC-KiTS, CPTCA-CCRCC, TCGA-KIRC, TCGA-KIRP, TCGA-KICH from The Cancer Imaging Archive (TCIA) [1] and there local-hospitals data from the eighth affiliated of Sun Yat-sen University ,Sun Yat-sen University Cancer Center ,Shenzhen Luohu People's Hospital are incorporated into this study. C4KC-KiTS includes 210 CT scan images and kidneys/tumor image segmentation results. For the data in TCGA, CPTCA and local hospitals, some images are disordered, low-quality chest or brain scans, postsurgical without kidney. Two well-trained clinical graduate students and two radiologists helped the data cleaning and filtering and cases with corticomedullary phase CT scan images containing renal regions were kept. In this study, we strictly abide by the following inclusion criteria: 1. According

to the new system[2], the goal of PN is reached when (1) surgical margins are negative, (2) WIT is <20 min, and (3) no major complications are observed(For samples from local hospitals, we strictly enforce this criterion, but the open-source data are filtered by urologists with reference to this criterion). 2. For the nephrographic phase, the CT values of blood in the abdominal aorta, renal cortex, and renal medulla are similar (The difference in CT value is less than or equal to 20HU). 3. For the corticomedullary phase, the CT value of blood in the abdominal aorta and the renal artery is greater than or equal to 250 HU. (To ensure sufficient contrast agent concentration). The renal cortex is significantly enhanced, and the renal medulla is slightly or not enhanced. The clinical, pathologic, and surgical of the TCGA cohort were collected from cBioportal [3]. To sum up, we collected 473 cases containing 473 corticomedullary phase CT images and related clinical pathologic.

CT image preparation and segmentation criteria

First of all, DICOM format primary files are transformed into NIfTI files by the dcm2niix tool [4]. Secondly, low-quality images, disorganized, postsurgical, or MRI images as well as the CT scan without abdominal area or enhanced images were cleaned and filtered by two well-trained clinical graduate students and two radiologists. Thirdly, 141 CT images and segmentation results from the C4KC-KiTS cohort are utilized to construct a kidney/tumor pre-segmentation model by a modified 2D Mask RCNN model [5; 6]. Fourthly, the proposed model is utilized to predict the

kidney/tumor segmentation of the 271 CT images from another dataset. AT last, two well-trained clinical graduate students and two radiologists manually mark the segmentation results by ITK-SNAP software [7]. After the above steps, 473 corticomedullary phase CT images and related kidney/tumor criteria segmentations are manufactured.

CT images feature extraction

Pyradiomics [8] module in PyPi is used to extract the kidney and tumor texture features, morphological features, and statistical features of the raw CT images with segmentations. For each corticomedullary phase CT scan image file, 200 features of the kidney and tumor region are extracted. Besides, we established two extractors about stage and grace and performed dimension reduction on the segmented voxels of CT images (Fig.3). First, we aligned the resolution of raw CT images to 2.0×1.5×1.5 mm. Secondly, the mean and standard deviation values of kidney and tumor regions are utilized to normalize all images. Thirdly, we calculated the central coordinate of the joint region of tumor and kidney. Fourthly, a region of 64×64×64 (12.8×9.6×9.6cm^3) only contained tumor and kidney is cropped with the center as the tumor and kidney center. The empty regions of the cropped images are filled with minimal pixel. Moreover, we constructed two feature extractors about stage and grace by 3D ResNet-18. It is an 18-layer CNN proposed, which are composed of 1 cross-layer connection and 17 stacked convolutional layers (Fig.S2) [9].

64 Deep Learning features from the cross-layer connection are extracted for each feature extractor. Lastly, singular value decomposition (SVD) and principal component analysis (PCA) are used to reduce the dimension of the cropped region into 64 and 256 by scikit-learn tools. SVD is a general method of decomposing a matrix into three deterministic matrices: $A = U\Sigma V^T$. With comparison of model performance, the best feature combination (Pyradiomics (200) + Deep Learning (64+64) +SVD (64) + PCA (256)) is introduced for prediction. In brief, a total of 648 features are used for classification of partial vs radical nephrectomy.

Machine learning model and algorithm

473 cases are randomly split into a training set (80%, 378 cases with 378 files) and a testing set (20%, 95 cases with 95 files). For image segmentation, the state-of-the-art model nnUNet [10] is used to construct kidney/tumor segmentation with a training set. Five-fold cross-validation is applied in the training process. Extreme gradient boosting (XGBoost [11], v1.3.3) was utilized to predict the surgical procedure of kidney tumor (partial versus radical nephrectomy) For the hyperparameter tuning in XGBoost, we utilized Grid Search to obtain the best value of following parameters: “min_child_weight”, “max_depth”, “n_estimators” and “max_depth”.

Model explaining with SHAP values

To evaluate the contribution of the imaging features to the model prediction, the SHapley Additive exPlanations (SHAP) [12] value of each feature for each sample was calculated. The SHAP values display the contribution to affect the predictive model of each feature. The shade of the dot indicates the different eigenvalue magnitudes, with redder dots designating larger eigenvalues and bluer dots designating lower eigenvalues. So, the top-20 ranks of mean absolute SHAP values show the features with great significance on classification.

Supplementary References

1 Clark K, Vendt B, Smith K et al (2013) The Cancer Imaging Archive (TCIA): maintaining and operating a public information repository. J Digit Imaging 26:1045-1057

2 Buffi N, Lista G, Larcher A, Lughezzani G, Ficarra V, Cestari A, Lazzeri M, Guazzoni G. Margin, ischemia, and complications (MIC) score in partial nephrectomy: a new system for evaluating achievement of optimal outcomes in nephron-sparing surgery. Eur Urol. 2012 Oct;62(4):617-8. doi: 10.1016/j.eururo.2012.06.001. Epub 2012 Jun 9. PMID: 22704367.

3 Cerami E, Gao J, Dogrusoz U et al (2012) The cBio cancer genomics portal: an open platform for exploring multidimensional cancer genomics data. Cancer Discov 2:401-404

4 Li X, Morgan PS, Ashburner J, Smith J, Rorden C (2016) The first step for neuroimaging data analysis: DICOM to NIfTI conversion. J Neurosci Methods 264:47-56

5 He K, Gkioxari G, Dollár P, Girshick R (2017) Mask r-cnnProceedings of the IEEE international conference on computer vision, pp 2961-2969

6 Jaeger PF, Kohl SA, Bickelhaupt S et al (2020) Retina U-Net: Embarrassingly simple exploitation of segmentation supervision for medical object detectionMachine Learning for Health Workshop. PMLR, pp 171-183

7 Yushkevich PA, Piven J, Hazlett HC et al (2006) User-guided 3D active contour segmentation of anatomical structures: significantly improved efficiency and

reliability. Neuroimage 31:1116-1128

8 van Griethuysen JJM, Fedorov A, Parmar C et al (2017) Computational Radiomics System to Decode the Radiographic Phenotype. Cancer Res 77:e104-e107

9 He K., Zhang X., Ren S., Sun J. Deep residual learning for image recognition; Proceedings of the IEEE Conference on Computer Vision and Pattern Recognition; Las Vegas, NV, USA. 27–30 June 2016; pp. 770–778

10 Isensee F, Jäger PF, Kohl SA, Petersen J, Maier-Hein KH (2019) Automated design of deep learning methods for biomedical image segmentation. arXiv preprint arXiv:190408128

11 Chen T, Guestrin C XGBoost: A scalable tree boosting system. arXiv 2016. arXiv preprint arXiv:160302754

12 Lundberg SM, Erion G, Chen H et al (2020) From Local Explanations to Global Understanding with Explainable AI for Trees. Nat Mach Intell 2:56-67
